# Culling in served females and farrowed sows at consecutive parities in Spanish pig herds

**DOI:** 10.1186/s40813-018-0080-y

**Published:** 2018-02-20

**Authors:** Satomi Tani, Carlos Piñeiro, Yuzo Koketsu

**Affiliations:** 1 School of Agriculture, Meiji University, Higashi-mita 1-1-1, Tama-ku, Kawasaki, Kanagawa 214-8571 Japan; 2 PigCHAMP pro Europa S.L., c/Santa Catalina 10, 40003 Segovia, Spain

**Keywords:** Culling risk, Lifetime data, Pigs born alive, Relative risk, Retention rate, Swine

## Abstract

**Background:**

The objectives of our study were 1) to characterize culling and retention patterns in parities 0 to 6 in served females and farrowed sows in two herd groups, and 2) to quantify the factors associated with by-parity culling risks for both groups in commercial herds. Lifetime data from first-service to removal included 465,947 service records of 94,691 females served between 2008 and 2013 in 98 Spanish herds. Herds were categorized into two groups based on the upper 25th percentile of the herd means of annualized lifetime pigs weaned per sow: high-performing (> 24.7 pigs) and ordinary herds (≤ 24.7 pigs). Two-level log-binomial regression models were used to examine risk factors and relative risk ratios associated with by-parity culling risks.

**Results:**

Mean by-parity culling risks (± SE) for served females and farrowed sows were 5.9 ± 0.03 and 12.4 ± 0.05%, respectively. Increased culling risks were associated with sows that farrowed 8 or fewer pigs born alive (PBA). Also, farrowed sows in high-performing herds in parities 2 to 6 had 1.5–5.6% higher culling risk than equivalent parity sows in ordinary herds (*P* < 0.05). Furthermore, sows in parities 1 to 6 that farrowed 3 or more stillborn piglets had 2.2–4.8% higher culling risk than for sows that did not farrow any stillborn piglets (*P* < 0.05). For served sows, culling risk in parity 1 to 6 sows with a weaning-to-first-service interval (WSI) of 7 days or more were 2.2–3.9% higher than equivalent parity sows with WSI 0–6 days (P < 0.05). With regard to relative risk ratios, served sows with WSI 7 days or more were 1.56–1.81 times more likely to be culled than those with WSI 0–6 days.

**Conclusion:**

Producers should reduce non-productive days by culling sows after weaning, instead of after service or during pregnancy. Also, producers should pay special attention to sows farrowing stillborn piglets or having prolonged WSI, and reconsider culling policy for mid-parity sows when they farrow 8 or fewer PBA.

**Electronic supplementary material:**

The online version of this article (10.1186/s40813-018-0080-y) contains supplementary material, which is available to authorized users.

## Background

Culling decisions in everyday practice are critical for retention patterns and financial performance in breeding herds. Annual culling rates are between 35.7 and 49.5% in the U.S.A., Spain, Sweden and Japan [1–3]. Furthermore, 30% of sows in commercial breeding herds can be culled by parity 3 [1]. Such low sow longevity decreases herd productivity and increases wastefulness of sow resources in the swine industry [4, 5]. At-risk groups for increased culling risk are low or high parity, sows that farrowed more stillborn piglets, and a high age of gilts at first-service [6, 7]. Major reasons for culling in low parities are reproductive failure and locomotor problems [8, 9]. A recent study showed that factors for culling risk due to reproductive failure in Japan are sows having prolonged weaning-to-first-service interval (WSI) and having farrowed fewer pigs born alive (PBA) [7]. Furthermore, it has been found that producers in high-performing herds in Japan cull more farrowed sows than producers in ordinary herds [2].

Culling policies for farrowed sows should be different from pregnant pigs in commercial herds. However, culling risk and retention patterns have not been well reported for either served pigs or farrowed sows in consecutive parities from 0 to 6. Also, there is little reported information about factors relating to culling risks for either served females or farrowed sows at consecutive parities, nor about lifetime records from first-served gilts to their removal.

It has been recommended that log-binomial regression modeling with relative risk ratios is a better method to use in cohort studies, rather than logistic regression modeling with odds ratios [10]. In this study, we will look at the relative risk ratios of culling in Spanish commercial breeding herds. However, no studies have been carried out of such Spanish herds with log-binomial regression models, despite Spain being a major pork producing country in Europe. Therefore, the specific objectives of the present study were 1) to characterize the pattern of retention and culling at consecutive parities for served females and farrowed sows by two herd groups, and 2) to quantify factors associated with by-parity culling risks for served females and farrowed sows, using log-binomial regression models.

## Methods

### Studied herds and data selection

A veterinary consultancy firm (PigCHAMP pro Europa S.L. Segovia, Spain) has requested all client producers to mail their data files on a regular basis and has accumulated a database. By the end of 2013, 98 Spanish client herds had allowed their herd data to be used for research purposes. The present study’s data, collected in 2013 with data for 2008–2013, included approximately 0.5% of all Spanish herds, with approximately 4% of female inventories in Spain; the country had 19,630 breeding herds and 2,568,450 females in December of 2013 [11].

Average herd size (± SEM) of the studied herds between 2008 and 2013 was 699 ± 64.3 females with a range between 81 and 3222 females. These studied herds used mechanical or natural ventilation in their breeding, gestation and farrowing barns. Their lactation and gestation diets were formulated using cereals (barley, wheat and corn) and soybean meal. Also, all the studied herds used artificial insemination; double or triple inseminations of sows during an estrous period are practiced for breeding management. Replacement gilts in the herds were either purchased from breeding companies or were home-produced through internal multiplication programs.

### Study design and exclusion criteria

The present study was designed as an epidemiological study coordinating reproductive data collected from the 98 Spanish herds. Gilts, first-served between 2008 and 2013, were observed until their removal or until the end of 2013 using the PigCHAMP recording system. When the data were collected, 4842 (4.9%) of the 99,533 sows had not yet been removed, and so they were excluded. Thus, the initial data contained 465,947 first-served records and 94,691 lifetime records in the 98 herds. Three datasets were created: Dataset 1 for calculating culling risks and retention rates, and Datasets 2 and 3 for log-binomial models of served females and farrowed sows, respectively. In Dataset 2, service records were omitted as missing records if they met any of the following criteria: total number of pigs born was 0 pigs or 26 pigs or more (817 records; [12]); lactation length was greater than 41 days (1966 records) and WSI is 36 days or more (4475 records; [13]). Also, when gilt age at first-service was examined, records were omitted if gilts had no record of age at first-service (3774 females), or if the recorded age of a gilt at first-service was either less than 160 days or more than 400 days (11,885 females; [13]). Hence, Dataset 2 comprised 465,947 first-served records in 94,691 females. Dataset 3 comprised the same records as Dataset 2 except for the exclusion of records for served females that were removed without farrowing (33,568 served records). Hence, Dataset 3 comprised 431,644 first-served and subsequently farrowed records of 87,752 sows.

### Definitions and categories

By-parity culling risk (%) for pigs served and sows farrowed were defined as the number of culled pigs divided by the number of female pigs served and sows farrowed, respectively, at that parity × 100. By-parity retention rate was defined as the number of sows that successfully reached farrowing at the next parity divided by the number of gilts first-served. A gilt was defined as a female pig that was entered into a herd but had not yet farrowed, and a sow was a female pig that had farrowed at least once.

To avoid the linearity assumption of the independent variable and to clearly describe an association, the herds were categorized into two herd groups, basis of the upper 25th percentile of the herd means of annualized lifetime pigs weaned per sow: high-performing herds (> 24.7 pigs) and ordinary herds (≤ 24.7 pigs). Herd size (± SEM) in high-performing and ordinary herds were 1095 ± 169 and 571 ± 59 females, respectively. Two WSI groups were formed: 0–6 days and 7 days or more.

Reasons for culling were recorded by producers when females were culled. Culling reasons were grouped into four categories: ‘reproductive failure,’ ‘lameness,’ ‘high parity’ and ‘others.’ Reproductive failure included no estrus, failure to farrow, found not pregnancy and abortion. The high parity culling category was restricted to sows of parity ≥4 at culling, because the median culling parity was 4, and so a planned culling for “high parity” could not be the reason for culling in a low parity [14]. Therefore, any low-parity sows recorded as being culled for high parity were regarded as missing records.

### Statistical analysis

Descriptive statistics were obtained using SAS version 9.3 (SAS Inst. Inc., Cary, NC). Dataset 1 was used for culling risks and retention rates. A log-binomial regression model was applied to the binary outcome in Dataset 2, i.e. whether or not a female pig was culled (1 or 0), by using the GLIMMIX procedure with a log link function with binomial distribution (DIST = BIN, LINK = LOG). The ILINK (inverse link function) was used to convert the logarithm to a probability [15]. Two-level analysis was applied to the models by using a herd as level 2 and an individual record as level 1. All the analyses were performed by parity in order to use the farrowed sow population at risk at each parity. In Dataset 2 for served gilts or sows, the following factors were assessed: for gilts models, age at first-service and the two herd groups; and for sows models, the two herd groups and two WSI groups. In Dataset 3 for farrowed sows, the following factors were assessed: the two herd groups, three PBA groups and three stillborn piglet groups. Also, a random herd effect and entry years were included in all models.

To select the most suitable final models, individual risk factors and possible interactions were examined. The model with the lowest pseudo-Akaike Information Criterion (pseudo-AIC) was selected as final models. The random effects for herds were obtained from residual log-pseudo-Likelihood using the COVTEST function. Additionally, the associations were considered significant when the *p*-value was <0.05. Pairwise multiple comparisons were performed using the Tukey-Kramer test.

### Intraclass correlation coefficient

The intraclass correlation coefficients (ICC) were calculated by the following eq. [16] to assess the variation in culling risk that could be explained by the herd:

$$ \mathrm{ICC}\left(\mathrm{records}\  \mathrm{within}\  \mathrm{the}\  \mathrm{same}\  \mathrm{herd}\right)={\sigma}_{\upnu}^2/\left({\sigma}_{\upnu}^2+\left({\pi}^2/3\right)\right), $$

in which ${\sigma}_{\upnu}^2$ is the between-herd variance and *π*^2^/3 is the assumed variance at the individual record level.

## Results

Descriptive statistics of lifetime performance and by-parity reproductive performance of sows are shown in Table 1. Annualized culling rate (± SEM) for removed females was 44.4 ± 0.08%, and the overall percentage of the first-served cohort gilts that were removed by culling was 85.4 ± 0.11%.

**Table 1:** Reproductive data for female pigs in 98 Spanish herds

Measurements			Range	
	N	Mean ± SEM	Minimum	Maximum
Lifetime records				
Parity at removal	94,691	4.6 ± 0.01	0	13
Parity at culling	80,845	4.7 ± 0.01	0	13
Annualized culling rate, %	94,691	44.4 ± 0.08	–	–
Percentage of culled females, %	94,691	85.4 ± 0.11	–	–
Gilt age at first-service^a^, days old	87,814	251.7 ± 0.15	160	400
Parity records				
Served parity	465,947	2.6 ± 0.01	0	12
Farrowed parity	431,644	3.6 ± 0.01	1	13
Number of pigs born alive^b^	371,252	12.1 ± 0.01	0	25
Number of stillborn piglets^b^	371,224	0.9 ± 0.01	0	19
Lactation length, days^b^	369,665	23.4 ± 0.01	0	41
Weaning-to-first-service interval, days^b^	367,502	5.9 ± 0.01	0	35

^a^The remaining records (94,691-N) were regarded as missing records

^b^The remaining records (465,947-N) were regarded as missing records

Table 2 shows the culling risks and retention rates of the first-served gilt cohorts at consecutive served and farrowed parities. Retention rates by parities 1, 2 and 3 were 92.7, 80.9 and 72.2%, respectively. Also, retention rates of served gilts decreased by 49.7% from parity 1 to 6. In particular, retention rate decreased by 19.1% from first-service at parity 0 to farrowing at parity 2. Mean by-parity culling risks (± SE) for served females and farrowed sows were 5.9 ± 0.03 and 12.4 ± 0.05%, respectively. Culling risks for served females decreased from 6.0% at parity 0 to 4.6% at parity 2, but then gradually increased to 8.0% by parity 6. Also, culling risks for farrowed sows increased rapidly from 4.0% at parity 2 to 39.0% at parity 7. Above parity 4, culling risks for farrowed sows were relatively higher than those for served females. For culling reasons, 38.9% of served females and 12.7% of farrowed sows were culled due to reproductive failure. In contrast, 9.7% of served females and 45.5% of farrowed sows were culled due to high parity.

**Table 2:** By-parity culling risks, farrowing rates and retention rates (%) of first-served female pigs

	Served parity (from service prior to subsequent farrowing)							
Measurements	0	1	2	3	4	5	6^b^	Total
Number of first-served female pigs	94,691	81,897	72,622	63,909	54,731	44,573	30,733	465,947
Re-served female pigs	11,947	9492	6086	5125	4080	2895	1733	42,442
Culled females without farrowing	5667	4300	3369	3390	3133	3028	2446	27,414
Dead females without farrowing	1272	981	926	911	909	800	598	6889
Re-service risks, %	12.6	11.6	8.4	8.0	7.5	6.5	5.6	9.1
Culling risks for pregnant pigs, %	6.0	5.3	4.6	5.3	5.7	6.8	8.0	29.0
	Farrowed parity (from farrowing prior to subsequent service)							
	1	2	3	4	5	6	7	Total
Number of sows farrowed in current parity^a^	87,752	76,616	68,327	59,608	50,689	40,745	27,689	431,644
Retention rates, %	92.7	80.9	72.2	63.0	53.5	43.0	29.2	–
Farrowing rates including re-served females, %	92.7	93.6	94.1	93.3	92.6	91.4	90.1	92.6
Culled females without subsequent service	4370	3095	3507	4097	5365	9050	10,809	53,431
Dead female records without subsequent service	1485	899	911	780	751	962	644	6957
Culling risks for farrowed sows, %	5.0	4.0	5.1	6.9	10.6	22.2	39.0	56.4

^a^Number of sows farrowed was calculated as the number of served records subtracted by the number of female pigs that died or were culled before farrowing

^b^Parity 7 or higher are not shown in Table 2 because these variables are similar to parity 6 sows

Tables 3, 4 and 5 shows the model selection for two-level mixed-effects log-binomial regression models for served females and farrowed sows. The final model was selected by the lowest pseudo-AIC in each model. A higher culling risk for served gilts was associated with increased age of gilts at first-service (Table 3; Additonal file 1: Appendix A), but the increase in culling risk was only 0.3% even when the age of gilts at first-service increased by 100 days. Furthermore, a decreased culling risk for served sows was associated with sows in high-performing herds in parity 2, and parities 4 to 6 (Table 4; Additonal file 1: Appendix B). In addition, a higher culling risk for served sows from parities 1 to 6 was associated with having WSI 7 days or more. With regard to the farrowed sows, higher culling risks for farrowed sows were associated with sows in high-performing herds in parities 2 to 6, sows farrowing 8 or fewer PBA, and sows farrowing 3 or more stillborn piglets in parities 1 to 6 (Table 5; Additonal file 1: Appendix C; *P* < 0.05).

**Table 3:** Model selection for two-level mixed-effects log-binomial regression models for served gilts

	Model specification				pseudo-AIC
Model for culling risk of served gilts					
	Fixed effect^a,b^			Random effect	Parity
Model No.	AFS	HG	Interaction	Herds	0
			AFS × HG		
1	✓			✓	39187^c^
2		✓		✓	41,896
3	✓	✓	✓	✓	NC
4	✓	✓		✓	39,189
COVTEST for final Model					<0.01

*AFS* age at first-service, *HG* herd groups

^a^Entry years were included into all models

^b^The univariate models were not shown because the AIC were higher than those shown in Models 1 and 2

^c^Final model is selected by the lowest pseudo-AIC

NC means the models did not converge

**Table 4:** Model selection for two-level mixed-effects log-binomial regression models for served sows

	Model specification				pseudo-AIC					
Model for culling risk of served sows										
	Fixed effect^a,b^			Random effect	Consecutive parities					
Model No.	HG	WSI	Interaction	Herds	1	2	3	4	5	6
			HG × WSI							
1	✓			✓	31,377	26,055	25,598	23,283	21,565	16,510
2		✓		✓	31207^c^	25,890	25,462	23,158	21,491	16,469
3	✓	✓	✓	✓	31,209	25887^c^	25,464	23,154	21,489	16,465
4	✓	✓		✓	31,209	25,889	25462^c^	23154^c^	21489^c^	16463^c^
COVTEST for final Model					<0.01	<0.01	<0.01	<0.01	<0.01	<0.01

*HG* herd groups, *WSI* weaning-to-first-service interval

^a^Entry years were included into all models

^b^The univariate models were not shown because the AIC were higher than those shown in Models 1 and 2

^c^Final model is selected by the lowest pseudo-AIC

**Table 5:** Model selection for two-level mixed-effects log-binomial regression models for farrowed sows

	Model specification							pseudo-AIC					
Model for culling risk of farrowed sows													
	Fixed effect^a,b^						Random effect	Consecutive parities					
Model No.	HG	PBA	SB	Interaction	Interaction	Interaction	Herds	1	2	3	4	5	6
				HG × PBA	HG × SB	PBA × SB							
1	✓						✓	34,280	24,293	26,657	28,691	32,820	39,007
2		✓					✓	34,062	23,631	25,841	27,850	31,659	NC
3			✓				✓	34,136	24,145	26,505	28,510	32,542	38,858
4	✓	✓	✓	✓	✓	✓	✓	33,970	23,516	25,717	27,716	31,472	37,658
5	✓	✓	✓	✓	✓		✓	33,976	23,511	25714^c^	27,713	31,488	37,672
6	✓	✓	✓		✓	✓	✓	33,967	23,534	25,729	27,727	31,484	NC
7	✓	✓	✓	✓		✓	✓	33,968	23,512	25,718	27,713	31469^c^	37655^c^
8	✓	✓	✓	✓			✓	33,974	23508^c^	25,715	27710^c^	31,486	37,670
9	✓	✓	✓		✓		✓	33,973	23,530	25,727	27,723	31,498	NC
10	✓	✓	✓			✓	✓	33,965	23,532	25,733	27,723	31,481	NC
11	✓	✓		✓			✓	34,067	23,611	25,818	27,835	31,646	NC
12		✓	✓			✓	✓	33964^c^	23,537	25,745	27,731	31,486	37,668
13	✓		✓		✓		✓	34,140	24,146	26,494	28,507	32,545	38,857
14	✓	✓	✓				✓	33,971	23,528	25,730	27,719	31,498	NC
COVTEST for Model								<0.01	<0.01	<0.01	<0.01	<0.01	<0.01

*HG* herd groups, *PBA* pigs born alive, *SB* stillborn piglets

^a^Entry years were included into all models

^b^The univariate models were not shown because the AIC were higher than those shown in Models 1–3

^c^Final model is selected by the lowest pseudo-AIC

NC means the models did not converge

Table 6 shows comparisons between herd groups and between WSI groups for culling risks and relative culling risk ratios for served sows in consecutive parities. Served sows in parities 4 to 6 in high-performing herds were 0.77–0.82 times as likely to be culled as those in ordinary herds (Table 6). Also, served sows with WSI 7 days or more were 1.56–1.81 times more likely to be culled than those with WSI 0–6 days. Culling risks for served sows in high-performing herds in parities 4 to 6 were 1.4–2.3% lower than those of sows in ordinary herds (*P* < 0.05). Also, the culling risks of sows in parities 1 to 6 that had WSI 7 days or more were 2.2–3.9% higher than equivalent parity sows which had WSI 0–6 days (*P* < 0.05). Furthermore, there was a two-way interaction for culling risk between the herd groups and WSI groups in parity 2. Sows with WSI 7 days or more in ordinary herds had 2.0% higher culling risk than those in high-performing herds (*P* < 0.05). However, there was no difference between the herd groups for culling risk in sows with WSI 0–6 days (*P* ≥ 0.05).

**Table 6:** Comparisons of culling risks (%) for served sows between either herd groups or weaning-to-first-service interval (WSI) groups, and the relative risk ratios for culling at different parities estimated by the models

	Parity 1	Parity 2	Parity 3	Parity 4	Parity 5	Parity 6
Final model No.	Model 2	Model 3	Model 4	Model 4	Model 4	Model 4
Groups	Culling risks (± SE), %					
Herd groups						
High-performing herds	–	4.1 (0.39)^b^	5.3 (0.42)	5.3 (0.45)^b^	6.8 (0.53)^b^	7.7 (0.64)^b^
Ordinary herds	–	5.2 (0.29)^a^	6.1 (0.30)	6.8 (0.36)^a^	8.2 (0.41)^a^	10.0 (0.53)^a^
WSI groups						
0–6 days	3.6 (0.19)^b^	3.5 (0.19)^b^	4.3 (0.20)^b^	4.5 (0.22)^b^	5.9 (0.26)^b^	7.0 (0.33)^b^
7 days or more	5.8 (0.32)^a^	6.1 (0.40)^a^	7.5 (0.44)^a^	8.1 (0.51)^a^	9.5 (0.59)^a^	10.9 (0.76)^a^
Groups	Relative risk ratio for culling (95% CI)					
Herd groups						
High-performing herds	–	NA	0.88 (0.73–1.05)	0.79 (0.65–0.95)	0.82 (0.69–0.98)	0.77 (0.64–0.93)
Ordinary herds	–	1.0	1.0	1.0	1.0	1.0
WSI groups						
0–6 days	1.0	1.0	1.0	1.0	1.0	1.0
7 days or more	1.60 (1.50–1.72)	NA	1.75 (1.61–1.91)	1.81 (1.65–1.99)	1.63 (1.47–1.80)	1.56 (1.39–1.75)

*SE* standard error, *CI* confidence interval

*NA* Relative risk ratios at parity 2 were not shown because there was a two-way interaction at parity 2

^a,b^Mean values within a column followed by different letters differ (*P* < 0.05)

Table 7 shows comparisons of herd, PBA and stillborn piglet groups for culling risks in farrowed sows at consecutive parities. Also, relative risk ratios were not shown in Table 7 because there were two-way interactions for culling risks in all the parity groups (Table 5). Culling risks for farrowed sows in high-performing herds in parities 2 to 6 were 1.5–5.6% higher than for equivalent parity sows in ordinary herds (*P* < 0.05). Also, sows that farrowed 8 or fewer pigs in parities 1 to 6 had 2.8–19.7% higher culling risks than for sows that farrowed 16 pigs or more (*P* < 0.05). Culling risks of sows that farrowed 3 or more stillborn piglets from parities 1 to 6 were 2.2–4.8% higher than for sows that did not farrow any stillborn piglets (*P* < 0.05).

**Table 7:** Comparisons of culling risks (%) for farrowed sows between either herd groups or stillborn piglet groups, and the relative risk ratios for culling at different parities estimated by the models^d^

	Parity 1	Parity 2	Parity 3	Parity 4	Parity 5	Parity 6
Final model No.	Model 12	Model 8	Model 5	Model 8	Model 7	Model 7
Groups	Culling risks (± SE)					
Herd groups						
High-performing herds	–	5.1 (0.53)^a^	7.0 (0.68)^a^	8.8 (0.94)^a^	12.4 (1.28)^a^	20.9 (2.78)^a^
Ordinary herds	–	3.6 (0.27)^b^	4.5 (0.31)^b^	6.2 (0.43)^b^	9.5 (0.63)^b^	15.3 (1.23)^b^
Pigs born alive groups						
16 or more	4.3 (0.44)^b^	2.5 (0.24)^c^	3.1 (0.26)^c^	4.4 (0.34)^c^	6.5 (0.51)^c^	11.4 (0.99)^c^
9–15	4.3 (0.23)^b^	3.3 (0.21)^b^	4.3 (0.25)^b^	5.6 (0.36)^b^	8.6 (0.53)^b^	16.2 (1.25)^b^
8 or fewer	7.1 (0.40)^a^	9.4 (0.62)^a^	13.1 (0.82)^a^	16.2 (1.09)^a^	23.0 (1.46)^a^	31.1 (2.42)^a^
Stillborn piglet groups						
0	4.3 (0.24)^b^	3.3 (0.22)^c^	4.6 (0.28)^b^	6.0 (0.40)^c^	9.2 (0.60)^b^	16.7 (1.32)^b^
1–2	4.7 (0.29)^b^	3.9 (0.27)^b^	5.0 (0.32)^b^	6.9 (0.46)^b^	9.9 (0.65)^b^	17.6 (1.39)^b^
3 or more	6.5 (0.62)^a^	6.1 (0.49)^a^	7.7 (0.54)^a^	9.7 (0.68)^a^	14.0 (1.02)^a^	19.4 (1.63)^a^

*SE* standard error

^a-c^Mean values within a column followed by different letters differ (*P* < 0.05)

^d^Relative risk ratios were not shown because there were two-way interactions in all parities

Table 8 shows the characteristics for the two-way interactions between the herd groups and PBA groups for culling risks of farrowed sows in parities 2 to 6. For instance, sows that farrowed 8 or fewer PBA in high-performing herds had 5.6–10.2% higher culling risks than those in ordinary herds (*P* < 0.05). But, there were no differences between herd groups for sows that farrowed 16 or more PBA (*P* ≥ 0.05). Table 9 also shows the characteristics for the two-way interactions between the PBA groups and stillborn piglet groups for culling risks of farrowed sows in parity 1 and parities 5–6. Across stillborn piglet groups, sows farrowing 8 or fewer PBA had 1.6–20.2% higher culling risks than sows that farrowed 16 or more PBA (*P* < 0.05). In contrast, across the PBA groups, sows that farrowed 3 or more stillborn piglets had 1.3–5.7% higher culling risks than sows that did not farrow any stillborn piglets (*P* < 0.05). With regard to the ICC, the random herd effect explained 3.0–11.1% of the total variance values for culling risks for served and farrowed females.

**Table 8:** Comparisons of culling risks (%) between herd groups and pigs born alive groups for farrowed sows in parities 2 to 6

	Pigs born alive groups		
	8 or fewer	9–15	16 or more
Herd groups	Culling risks (± SE), %		
	Parity 2		
High-performing herds	12.6 (1.36)^ax^	3.7 (0.39)^y^	2.8 (0.38)^y^
Ordinary herds	7.0 (0.52)^bx^	3.0 (0.21)^y^	2.3 (0.30)^y^
	Parity 3		
High-performing herds	18.2 (1.87)^ax^	5.2 (0.51)^ay^	3.6 (0.42)^z^
Ordinary herds	9.4 (0.67)^bx^	3.6 (0.23)^by^	2.7 (0.31)^y^
	Parity 4		
High-performing herds	21.3 (2.38)^ax^	6.8 (0.73)^ay^	4.7 (0.56)^z^
Ordinary herds	12.3 (0.91)^bx^	4.7 (0.32)^by^	4.1 (0.41)^y^
	Parity 5		
High-performing herds	28.7 (3.06)^ax^	10.0 (1.03)^y^	6.6 (0.78)^z^
Ordinary herds	18.5 (1.26)^bx^	7.4 (0.48)^y^	6.3 (0.61)^y^
	Parity 6		
High-performing herds	36.1 (4.82)^x^	19.9 (2.63)^y^	12.7 (1.78)^z^
Ordinary herds	26.8 (2.14)^x^	13.2 (1.04)^y^	10.2 (1.00)^z^

^a,b^Mean values within a column followed by different letters differ (*P* < 0.05)

^x-z^Mean values within a row followed by different letters differ (*P* < 0.05)

**Table 9:** Comparisons of culling risks (%) between pigs born alive groups and stillborn piglets groups for farrowed sows in parities 1 and 5–6

	stillborn piglets groups		
	0	1–2	3 or more
Pigs born alive groups	Culling risks (± SE), %		
	Parity 1		
8 or fewer	5.7 (0.37)^ay^	5.9 (0.43)^ay^	10.5 (0.74)^ax^
9–15	3.7 (0.20)^by^	4.0 (0.23)^by^	5.2 (0.39)^bx^
16 or more	3.7 (0.34)^b^	4.3 (0.46)^ab^	5.0 (1.20)^b^
	Parity 5		
8 or fewer	21.6 (1.48)^ay^	20.7 (1.54)^ay^	27.3 (1.93)^ax^
9–15	6.8 (0.44)^bz^	7.8 (0.50)^by^	11.9 (0.81)^bx^
16 or more	5.3 (0.48)^cy^	6.1 (0.55)^cxy^	8.4 (1.12)^bx^
	Parity 6		
8 or fewer	30.5 (2.42)^a^	30.4 (2.43)^a^	32.4 (2.56)^a^
9–15	14.4 (1.13)^bz^	15.8 (1.24)^by^	18.6 (1.48)^bx^
16 or more	10.7 (0.99)^c^	11.3 (1.05)^c^	12.2 (1.51)^c^

^a-c^Mean values within a column followed by different letters differ (*P* < 0.05)

^x-z^Mean values within a row followed by different letters differ (*P* < 0.05)

## Discussion

Our study showing a 72% retention rate at parity 3 is within the range reported in previous studies in Sweden and Japan of 70 and 77%, respectively [1, 17]. In particular, 4.6 to 6.0% of served females were culled from parities 0 to 2 in our study. This culling of pregnant gilts and sows increases non-productive days, and decreases herd profitability because positive net income for each sow is not obtained until parity 3 [18]. Additionally, approximately 10% of served females return to estrus, and 33% of the returned females have another return [19, 20]. Furthermore, low parity females are likely to become severe repeat-breeders with three or more returns [21]. So it is necessary for producers to perform frequent estrus checks for served gilts and sows in the first 3–6 weeks post service to minimize non-productive days because 60% of female returns occur during such periods [20].

More farrowed sows are culled due to high age than served females, but in contrast more served females are culled due to reproductive failure than farrowed sows. In fact, our study shows that approximately 40% of the served gilts and sows were culled due to reproductive failure, without any successful farrowing. A Swedish report showed only 26.9% of culling was due to reproductive failure [1], so our studied herds may have had more culling due to reproductive failure than voluntary culling. Risk factors in the U.S.A. are low parity, summer season, short lactation length and lower lactation feed intake [22].

Our study also showed 1.4–2.3% lower culling risk for served sows from parities 4 to 6 in high-performing herds than those in ordinary herds. This can be explained by higher farrowing rates, lower return risks, and better management for pregnant sows in high-performing herds [2, 23, 24]. Also, the fact that there were no differences between the herd groups in culling risk for pregnant pigs in parity 3 and for sows having WSI 0–6 days in parity 2, indicates that there was little difference in culling policy implemented between the two herd groups for served or pregnant pigs in low to mid parity or for sows with WSI 0–6 days that are expected to become a good sow [25].

High-performing herds appear to relentlessly cull farrowed sows from parity 2 or higher without subsequent service. Culling risks in farrowed sows in parities 2 to 6 were 1.5–5.6% higher in high-performing herds than in ordinary herds. This is because it is better to cull farrowed sows without subsequent service rather than served sows, because culling pregnant sows increases culling interval and non-productive days.

Our present study showing a higher culling risk in sows that farrowed 8 or fewer PBA than in sows that farrowed 16 or more PBA is consistent with a previous study in Japan, and shows that producers tend to cull sows that farrowed few PBA [7]. It appears that Spanish high-performing herds cull parity 2 to 5 sows that farrowed 8 or fewer PBA more than ordinary herds. However, a previous study in Japan showed that sows in parities 4 and 5 still have higher farrowing rates and more PBA than incoming gilts [26]. So, it is recommended that sows in parities 2 to 4 that farrow 8 or fewer PBA should not be culled unless they have reproductive failure, locomotor problems, or if the herd does not have space to house such sows, or aims to rapidly change genetics.

The increased culling risks in served sows with WSI 7 days or more in our present study correspond with previous studies showing a high culling risk in sows with prolonged WSI [7, 27]. Sows with prolonged WSI have lower farrowing rates and fewer PBA than sows with WSI 0–6 days [25, 28]. So, producers appear to cull sows that are expected to have low reproductive performance at subsequent parity. The WSI is affected by gonadotropin secretion from the hypothalamic-pituitary-gonadal axis, which is reduced if sow feed intake is decreased during lactation [29, 30]. In order to reduce the number of sows having a prolonged WSI, feed consumption should be increased for lactating sows [22]. Therefore, it is recommended that producers pay attention to cooling management during the summer season and good nutritional management such as increased feed consumption for lactational sows.

In our study, the higher culling risks for sows that farrowed 3 or more stillborn piglets compared with sows that farrowed 0 stillborn piglets could be explained by an increased likelihood of infectious events or manual intervention during farrowing difficulties [31]. Sows that farrow more stillborn piglets have increased return risks in late gestation or higher abortion risk [20, 32]. Therefore, special treatments should be considered for sows that farrow many stillborn piglets or that have dystocia. Finally, gilt age at first-service is not very important as a risk factor because there was only a 0.3% increased risk per 100-day increased gilt age. With regard to the relatively high ICC for herd variance of 3.0–11.1%, there were some effects of the herd on culling, such as management or production systems.

To our knowledge, this is the first study applying log-binomial regression models with relative risk ratios to culling risks of female pigs in commercial herds. The convergence failure in our parity 6 models can be explained by the fact that a log-binomial model is less stable than logistic models, and there have been some cases where log-binomial models failed to converge [10].

Finally, there are some limitations that should be noted when interpreting the results of this observational study using commercial herd data. Our studied herds were not randomly selected from all Spanish herds. Also, our analyses did not take account of health status, nutritional programs, genotype or housing types. However, even with such limitations, this research provides valuable information about culling and retention patterns, and the quantitative relationship between production factors and culling risks that should help swine producers and practicing veterinarians to maximize their sows’ reproductive potential.

## Conclusion

To achieve high retention in low parity and improve longevity, it is recommended that producers provide appropriate management for sows farrowing stillborn piglets or having prolonged WSI. Also, culling policy in all herds should be reconsidered for parity 2 to 4 sows that farrowed 8 or fewer PBA. Finally, to reduce non-productive days, producers should cull sows after weaning, not after service or during pregnancy.

## Additional file

Additional file 1: Appendix A) Estimates of factors in the log-binomial regression model for culling risks of served gilts. Appendix B) Estimates of factors in the log-binomial regression models for culling risks of served sows. Appendix C) Estimates of factors in the log-binomial regression models for culling risks of farrowed sows. (DOCX 94 kb)

## Abbreviations

ICC: Intraclass correlation coefficients
PBA: Pigs born alive
pseudo-AIC: Pseudo-Akaike Information Criterion
WSI: Weaning-to-first-service interval

## References

[1] Engblom L, Lundeheim N, Dalin A-M, Andersson K (2007). Sow removalin Swedish commercial herds. Livest Sci.

[2] Sasaki Y, Koketsu Y (2010). Culling intervals and culling risks in four stages of the reproductive life of first service and reserviced female pigs in commercial herds. Theriogenology.

[3] PigCHAMP. PigCHAMP benchmarks. 2011. http://www.pigchamp.com/Portals/0/Documents/Benchmarking%20Summaries/USA%202011.pdf. Accessed 30 Aug 2017.

[4] Koketsu Y (2007). Longevity and efficiency associated with age structures of female pigs and herd management in commercial breeding herds. J Anim Sci.

[5] Stalder K, D’Allaire S, Drolet R, Abell C, Zimmerman JJ, Karriker LA, Ramirez A, Schwartz KJ, Stevenson GW (2012). Longevity in breeding animals. Deseases of swine.

[6] Zhao Y, Liu X, Mo D, Chen Q, Chen Y (2015). Analysis of reasons for sow culling and seasonal effects on reproductive disorders in southern China. Anim Reprod Sci.

[7] Tani S, Koketsu Y (2016). Factors for culling risk due to pregnancy failure in. breeding-female pigs. J Agric Sci.

[8] Sasaki Y, Koketsu Y (2011). Reproductive profile and lifetime efficiency of female pigs by culling reason in high-performing commercial breeding herds. J Swine Health Prod.

[9] Tinkle AK, Duberstein KJ, Wilson ME, Parsley MA, Beckman MK, Torrison J, Azaina MJ, Dove CR (2017). Functional claw trimming improves the gait and locomotion of sows. Livest Sci.

[10] Spiegelman D, Hertzmark E (2005). Easy SAS Calculations for risk or prevalence ratios and differences. Am J Epidemiol.

[11] European commission. Pig: number of farms and heads by agricultural size of farm (UAA) and size of pig herd, 2016. http://ec.europa.eu/eurostat/web/products-datasets/-/ef_lspigaa. Accessed 30 Aug 2017.

[12] Lundgren H, Canario L, Grandinson K, Lundeheim N, Zumbach B, Vangen O, Rydhmer L (2010). Genetic analysis of reproductive performance in landrace sows and its correlation to piglet growth. Livest Sci.

[13] Hoving L, Soede N, Graat E, Feitsma H, Kemp B (2011). Reproductive performance of second parity sows: relations with subsequent reproduction. Livest Sci.

[14] Andersson E, Frössling J, Engblom L, Algers B, Gunnarsson S (2016). Impact of litter size on sow stayability in Swedish commercial piglet producing herds. Acta Vet Scand.

[15] Littell RC, Milliken GA, Stroup WW, Wolfinger RD, Schabenberger O (2006). SAS for mixed models second edition.

[16] Dohoo IR, Martin SW, Stryhn H (2009). Veterinary epidemiologic research.

[17] Iida R, Kaneko M, Koketsu Y. Low retention rate of female pigs associated with gilt development, lifetime performance and culling pattern in commercial swine herds. 2015.

[18] Sasaki Y, McTaggart I, Koketsu Y (2011). Assessment of lifetime economic returns of sows by parity of culled sows in commercial breeding herds. J Vet Epidemiol.

[19] Vargas AJ, Bernardi ML, Bortolozzo FP, Mellagi APG, Wentz I (2009). Factors associated with return to estrus in first service swine females. Prev Vet Med.

[20] Tani S, Piñeiro C, Koketsu Y (2016). Recurrence patterns and factors associated with regular, irregular, and late return to service of female pigs and their lifetime performance on southern European farms. J Anim Sci.

[21] Tani S, Piñeiro C, Koketsu Y (2017). Characteristics and risk factors for severe repeat-breeder female pigs and their lifetime performance in commercial breeding herds. Porcine Health Management.

[22] Koketsu Y, Dial GD, Pettigrew JE, King VL (1996). Feed intake pattern during lactation and subsequent reproductive performance of sows. J Anim Sci.

[23] Stein TE, Duffy SJ, Wickstrom S (1990). Differences in production values between high- and low productivity swine breeding herds. J Anim Sci.

[24] King VL, Koketsu Y, Reeves D, Xue JL, Dial GD (1998). Management factors associated with swine breeding-herd productivity in the United States. Prev Vet Med.

[25] Tummaruk P, Tantasuparuk W, Techakumphu M, Kunavongkrit A (2010). Influence of repeat-service and weaning-to-first-service interval on farrowing proportion of gilts and sows. Prev Vet Med.

[26] Iida R, Koketsu Y (2016). Lower farrowing rate in female pigs associated with higher outdoor temperatures in humid subtropical and continental climate zones in Japan. Anim Reprod Belo Horizonte.

[27] Bertoldo MJ, Grupen CG, Thomson PC, Evans G, Holyoake PK (2009). Identification of sow-specific risk factors for late pregnancy loss during the seasonal infertility period in pigs. Theriogenology.

[28] Koketsu Y (1999). Assessment of sows mating efficacy during the low productive period after early weaning: a field study. Theriogenology.

[29] Koketsu Y, Dial GD, Pettigrew JE, Marsh WE, King VL (1996). Influence of imposed feed intake patterns during lactation on reproductive performance and on circulating levels of glucose, insulin, and luteinizing hormone in primiparous sows. J Anim Sci.

[30] Soede NM, Langendijk P, Kemp B (2011). Reproductive cycles in pigs. Anim Reprod Sci.

[31] Almond GW, Flowers WL, Batista L, D’Allaire S, Straw BE, Zimmerman JJ, D’Allaire S, Taylor DJ (2006). Diseases of the reproductive system. Diseases of swine.

[32] Iida R, Piñeiro C, Koketsu Y (2016). Abortion occurrence, repeatability and factors associated with abortions in female pigs in commercial herds. Livest Sci.

