# Supplementary material for: Culling in served females and farrowed sows at consecutive parities in Spanish pig herds
Source: Porcine Health Manag. 2018 Feb 20;4:3. doi: 10.1186/s40813-018-0080-y (PMC5819191; doi:10.1186/s40813-018-0080-y)
Supplement: Supplementary file 1 — Appendix A) Estimates of factors in the log-binomial regression model for culling risks of served gilts. Appendix B) Estimates of factors in the log-binomial regression models for culling risks of served sows. Appendix C) Estimates of factors in the log-binomial regression models for culling risks of farrowed sows. (DOCX 94 kb) [file 40813_2018_80_MOESM1_ESM.docx]

**Appendix A.** Estimates of factors in the log-binomial regression model for culling risks of served gilts

| Parity (Final model No.) | Parity 0 (Model 1) |
| --- | --- |
| Fixed and random effects^1^ | Estimate (± SE) |
| Intercept | - 3.006 (0.058)^2^ |
| Gilt age at first-service | 0.003 (0.001)^2^ |
| Herd variance | 0.2 (0.04) |
| ICC (records within the same herd) | 6.6 |

^1^SE: standard error; ICC: intraclass correlation coefficient.

^2^ indicates significant differences at the P < 0.05 level compared to 0.

**Appendix B.** Estimates of factors in the log-binomial regression models for culling risks of served sows

| Parity (Final model No.) | Parity 1 (Model 2) | Parity 2 (Model 3) | Parity 3 (Model 4) |
| --- | --- | --- | --- |
| Fixed and random effects^1^ | Estimate (± SE) | Estimate (± SE) | Estimate (± SE) |
| Intercept | - 3.298 (0.057)^2^ | - 3.146 (0.062)^2^ | - 2.970 (0.055)^2^ |
| High-performing herds | - | - 0.131 (0.107)^3^ | - 0.134 (0.091) |
| WSI 7 days or more | 0.472 (0.035)^4^ | 0.669 (0.052)^4^ | 0.562 (0.045)^4^ |
| High-performing herds x WSI 7 days or more | - | - 0.198 (0.096)^†^ | - |
| Herd variance | 0.2 (0.03) | 0.2 (0.03) | 0.1 (0.02) |
| ICC (records within the same herd) | 5.5 | 4.5 | 3.3 |
|  | Parity 4 (Model 4) | Parity 5 (Model 4) | Parity 6 (Model 4) |
| Fixed and random effects^1^ | Estimate (± SE) | Estimate (± SE) | Estimate (± SE) |
| Intercept | - 2.832 (0.058)^2^ | - 2.603 (0.055)^2^ | - 2.296 (0.059)^2^ |
| High-performing herds | - 0.238 (0.097)^3^ | - 0.193 (0.088)^3^ | - 0.262 (0.094)^3^ |
| WSI 7 days or more | 0.593 (0.048)^4^ | 0.486 (0.051)^4^ | 0.444 (0.059)^4^ |
| Herd variance | 0.1 (0.03) | 0.1 (0.02) | 0.1 (0.02) |
| ICC (records within the same herd) | 3.7 | 3.0 | 3.3 |

^1^SE: standard error; WSI: weaning-to-first-service interval; ICC: intraclass correlation coefficient.

^2^ indicates significant differences at the P < 0.05 level compared to 0.

^3,4^ indicates significant differences at the P < 0.05 level compared to the reference value (ordinary herds^3^ or WSI 0-6 days^4^).

^†^There was a two-way interaction.

**Appendix C.** Estimates of factors in the log-binomial regression models for culling risks of farrowed sows

| Parity (Final model No.) | Parity 1 (Model 12) | Parity 2 (Model 8) | Parity 3 (Model 5) |
| --- | --- | --- | --- |
| Fixed and random effects^1^ | Estimate (± SE) | Estimate (± SE) | Estimate (± SE) |
| Intercept | - 3.236 (0.093)^2^ | - 3.965 (0.135)^2^ | - 3.701 (0.116)^2^ |
| High-performing herds | - | 0.217 (0.188)^3^ | 0.344 (0.162)^3^ |
| Pigs born alive groups |  |  |  |
| 9-15 | 0.009 (0.081) | 0.277 (0.122)^4^ | 0.269 (0.101)^4^ |
| 8 or fewer | 0.432 (0.089)^4^ | 1.122 (0.127)^4^ | 1.233 (0.107)^4^ |
| Stillborn piglet groups |  |  |  |
| 1-2 | 0.138 (0.122) | 0.168 (0.040)^5^ | 0.164 (0.052)^5^ |
| 3 or more | 0.297 (0.246) | 0.614 (0.056)^5^ | 0.537 (0.069)^5^ |
| Herd groups x Pigs born alive groups |  |  |  |
| High-performing herds x 9-15 pigs | - | - 0.007 (0.156)^†^ | 0.092 (0.125)^†^ |
| High-performing herds x 8 pigs or fewer | - | 0.369 (0.162) | 0.384 (0.134) |
| Herd groups x Stillborn piglet groups |  |  |  |
| High-performing herds x 1-2 pigs | - | - | - 0.157 (0.073) |
| High-performing herds x 3 pigs or more | - | - | - 0.032 (0.095) |
| Pigs born alive x Stillborn piglet groups |  |  |  |
| 9-15 pigs x 1-2 pigs | - 0.070 (0.128)^†^ | - | - |
| 9-15 pigs x 3 or more | 0.027 (0.253) | - | - |
| 8 pigs or fewer x 1-2 pigs | - 0.102 (0.141) | - | - |
| 8 pigs or fewer x 3 pigs or more | 0.315 (0.255) | - | - |
| Herd variance | 0.2 (0.04) | 0.2 (0.04) | 0.2 (0.04) |
| ICC (records within the same herd) | 5.5 | 6.1 | 5.4 |
|  | Parity 4 (Model 8) | Parity 5 (Model 7) | Parity 6 (Model 7) |
| Fixed and random effects^1^ | Estimate (± SE) | Estimate (± SE) | Estimate (± SE) |
| Intercept | - 3.310 (0.104)^2^ | - 2.885 (0.104)^2^ | - 2.341 (0.103)^2^ |
| High-performing herds | 0.137 (0.156)^3^ | 0.040 (0.148)^3^ | 0.222 (0.169)^3^ |
| Pigs born alive groups |  |  |  |
| 9-15 | 0.133 (0.082) | 0.122 (0.087) | 0.208 (0.071)^4^ |
| 8 or fewer | 1.106 (0.088)^4^ | 1.206 (0.092)^4^ | 1.013 (0.073)^4^ |
| Stillborn piglet groups |  |  |  |
| 1-2 | 0.144 (0.034)^5^ | 0.135 (0.096) | 0.062 (0.075)^5^ |
| 3 or more | 0.483 (0.041)^5^ | 0.456 (0.137)^5^ | 0.135 (0.111)^5^ |
| Herd groups x Pigs born alive groups |  |  |  |
| High-performing herds x 9-15 pigs | 0.246 (0.106)^†^ | 0.256 (0.097)^†^ | 0.189 (0.076)^†^ |
| High-performing herds x 8 pigs or fewer | 0.409 (0.115) | 0.397 (0.103) | 0.077 (0.079) |
| Pigs born alive x Stillborn piglet groups |  |  |  |
| 9-15 pigs x 1-2 pigs | - | 0.001 (0.103)^†^ | 0.034 (0.078)^†^ |
| 9-15 pigs x 3 or more | - | 0.103 (0.144) | 0.118 (0.115) |
| 8 pigs or fewer x 1-2 pigs | - | - 0.179 (0.112) | - 0.063 (0.080) |
| 8 pigs or fewer x 3 pigs or more | - | - 0.225 (0.147) | - 0.072 (0.114) |
| Herd variance | 0.2 (0.05) | 0.2 (0.04) | 0.4 (0.06) |
| ICC (records within the same herd) | 6.9 | 6.7 | 11.0 |

^1^SE: standard error; ICC: intraclass correlation coefficient.

^2^ indicates significant differences at the P < 0.05 level compared to 0.

^3-5^ indicates significant differences at the P < 0.05 level compared to the reference value (ordinary herds^3^, 16 or more pigs born alive^4^ or 0 stillborn piglets^5^).

^†^There was a two-way interaction.
